# An Integrated and Multi-Target Nucleic Acid Isothermal Analysis System for Rapid Diagnosis of Vulvovaginal Candidiasis

**DOI:** 10.3390/bios13050559

**Published:** 2023-05-19

**Authors:** Xiangyu Jin, Meng Li, Zeyin Mao, Anni Deng, Wenqi Lv, Leyang Huang, Hao Zhong, Han Yang, Lei Zhang, Qinping Liao, Guoliang Huang

**Affiliations:** 1 Department of Biomedical Engineering, School of Medicine, Tsinghua University, Beijing 100084, China; 2 Department of Obstetrics and Gynecology, Beijing Tsinghua Changgung Hospital, School of Clinical Medicine, Tsinghua University, Beijing 102218, Chinazla00969@btch.edu.cn (L.Z.); lqpa00594@btch.edu.cn (Q.L.); 3 National Engineering Research Center for Beijing Biochip Technology, Beijing 102206, China

**Keywords:** loop-mediated isothermal amplification, vulvovaginal candidiasis, integrated detection, microfluidic chip

## Abstract

Rapid identification of *Candida* species is significant for the diagnosis of vulvovaginal candidiasis (VVC). An integrated and multi-target system for the rapid, high-specificity, and high-sensitivity detection of four *Candida* species was developed. The system consists of a rapid sample processing cassette and a rapid nucleic acid analysis device. The cassette could process the *Candida* species to release nucleic acids in 15 min. The released nucleic acids were analyzed by the device as fast as within 30 min, using the loop-mediated isothermal amplification method. The four *Candida* species could be simultaneously identified, with each reaction using only 1.41 µL of reaction mixture, which was low cost. The RPT (rapid sample processing and testing) system could detect the four *Candida* species with high sensitivity (<2 CFU/reaction) and high specificity. The system also processed and analyzed 32 clinical samples, giving the results with high clinical sensitivity and specificity. Hence, the system was a significant and effective platform for the diagnosis of VVC. Furthermore, the period of validity of the reagents and chips used in the system was >90 days, and the system could also be used for the detection of bacteria.

## 1. Introduction

Vulvovaginal candidiasis (VVC) is the second most common cause of vaginitis symptoms (after bacterial vaginosis), characterized by the symptoms of vulvovaginal inflammation and the presence of *Candida* species [1,2,3]. It is estimated that approximately 75% of healthy adult women experience at least one episode of VVC during their lives, with half of those infected experiencing the disease again [4,5]. The typical syndromes of VVC are vulvar pruritis, along with vulvar burning, irritation, soreness, and abnormal vaginal discharge [6,7,8]. The syndromes can also be dysuria or dyspareunia [7]. *Candida albicans* (*C. albicans*) is the most commonly identified *Candida* species in the vagina of VVC patients, accounting for approximately 70–90% VVC cases [3,6]. However, it is noteworthy that the frequency of the isolation of non-*albicans* species, especially *Candida glabrata* (*C. glabrata*), in VVC is increasing [9,10,11]. Other non-*albicans* species include *Candida parapsilosis* (*C. parapsilosis*), *Candida tropicalis* (*C. tropicalis*), and *Candida krusei* (*C. krusei*) [1,12,13].

Aside from the evaluation of the symptoms, the confirmation of the presence of *Candida* species is important for the diagnosis of VVC [14]. Microscopic examination, such as examining wet mount or Gram stain of the vaginal discharge, is commonly used to detect *Candida* species [14,15]. Such microscopic examination is low-cost and rapid, but the sensitivity is low if the specimens are not of high quality, or the clinical scientists are not experienced [16]. In contrast, fungal culturing can identify *Candida* species with high sensitivity and specificity and is normally thought of as the gold standard [7,17,18,19]. However, fungal culturing takes a long time (at least 1–2 days) and is laborious [20]. Molecular diagnostic methods, such as PCR, often have a faster turnaround than fungal culturing, along with high sensitivity and specificity [21,22]. Nevertheless, PCR relies on sophisticated and expensive instruments and requires a laboratory environment, making it unsuitable to be directly used in resource-limited regions [23,24].

Loop-mediated isothermal amplification (LAMP) is a rapid and isothermal amplification method [25]. It has been utilized for the detection of pathogens, such as SARS-CoV-2, and *methicillin-resistant Staphylococcus aureus* (MRSA), in POCT or resource-limited regions because it does not require sophisticated instruments [26,27]. There has been some work using LAMP to identify *Candida* species in an isothermal and multi-target way [28,29,30,31,32]. Some works also realized the detection with the participation of microfluidic chips [33,34]. However, none of them were involved in identifying different *Candida* species in clinical samples of VVC, especially *C. glabrata*, *C. parapsilosis*, and *C. tropicalis*. Furthermore, these reports processed the samples with commercial kits or traditional methods to obtain pure genomic DNAs for detection, which is time-consuming [33,34,35,36]. Hence, it would be significant to provide a platform that can identify the *Candida* species in clinical samples in a rapid, integrated, and automatic way to support the diagnosis of VVC, giving full play to the advantages of high efficiency of LAMP.

In this work, we reported an integrated and multi-target RPT system (rapid sample processing and testing system) for the rapid diagnosis of VVC. The RPT system consists of a rapid sample processing cassette and a rapid nucleic acid analysis device, realizing the automatically processing and detecting of *Candida*. Four *Candida* species (*C. albicans*, *C. glabrata*, *C. parapsilosis*, and *C. tropicalis*) were chosen as the detection targets. The cultured *Candida* samples were used to verify the performance of the RPT system. As a contrast, the cultured *Candida* samples were also processed and analyzed by commercial sample processing kits and real-time PCR. Moreover, clinical samples from 32 patients were also processed and analyzed by the RPT system to verify its clinical sensitivity and specificity. The period of the validity of the reagents and chips used in the RPT system and the feasibility of using the RPT system for bacterial detection (*Staphylococcus aureus* and *Pseudomonas aeruginosa*) were also demonstrated in this work.

## 2. Materials and Methods

### 2.1. Structure and Working Principle of the RPT System

**Figure 1:**
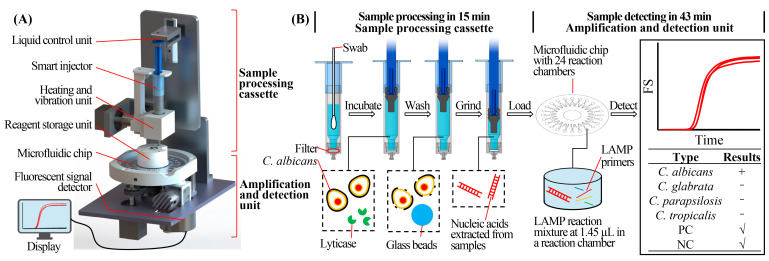
Schematic and working principle of the RPT system. (**A**) Schematic of the RPT system. (**B**) Working principle of the RPT system. RPT system, rapid sample processing and testing system; LAMP, loop-mediated isothermal amplification; FS, fluorescence signal; PC, positive control; NC, negative control.

The portable RPT system consists of a sample processing cassette and a nucleic acid analysis device (Figure 1A). The real images of the system are shown in Figure S1. The system could be powered by mains line or battery. It could automatically complete the analyzation after the insertion of a vaginal swab. The sample processing cassette consists of a smart injector, liquid control unit, heating and vibration unit, and reagent storage unit, as shown in Figure 1A. Lysis buffer (600 µL), consisting of 1 M sorbitol (Solarbio, China), 100 mM of EDTA (Solarbio, China), and 200 U of lyticase (Solarbio, China) in 1× PBS (Solarbio, China), was preloaded in the smart injector, which could precisely transfer liquid. A swab containing microorganisms was inserted into the lysis buffer, followed by the elution of microorganisms through vibration. Then, the swab was discarded, and the injector was assembled onto the liquid control unit. The lysis buffer with microorganisms was incubated at 30 °C for 10 min to lyse the *Candida* cell walls (Figure 1B) [37]. Then, the liquid control unit and smart injector discarded the lysis buffer and washed the microorganisms with TE buffer, which was prestored in the reagent storage unit. The injector was precisely controlled by the liquid control unit when aspirating and dispensing reagents. Microorganisms were reserved in the cassette due to the membrane filters fixed at the bottom of the smart injector (Figure 1B). Microorganisms were then resuspended in 100 µL TE buffer and mixed with glass beads (diameter = 100 µm; EASYBIO, China; prestored in the injector) for the grinding step by vibrating at 99 °C for 5 min to break the cells and release nucleic acids. The released nucleic acids could be used for amplification and detection.

The nucleic acid analysis device consists of a chip heating unit, a stepper motor, and a fluorescent signal detector. The multiplex targets could be analyzed in the microfluidic chip. The stepper motor controlled the rotation of the chip, realizing the scanning of the fluorescence signal of reaction chambers in the chip. The fluorescent signal detector could release excitation light, causing the production of emitted fluorescent light from the fluorescent dye combined with amplicon. The emitted light could also be collected and transformed into electrical signal by the detector for the subsequent recording and analyzing. The detailed structure and working principle of the detector are described in Section 1 of Supplementary Materials. The detector was also used in our previous work [29,38]. Each reaction chamber could be detected by the detector each minute. The real time curve of fluorescent signal was recorded and displayed by the corresponding software, as the Tt (Threshold time) was chosen to be analyzed for the test results. Finally, the RPT system reported the detection results on the display.

### 2.2. Structure and Working Principle of the Microfluidic Chip

**Figure 2:**
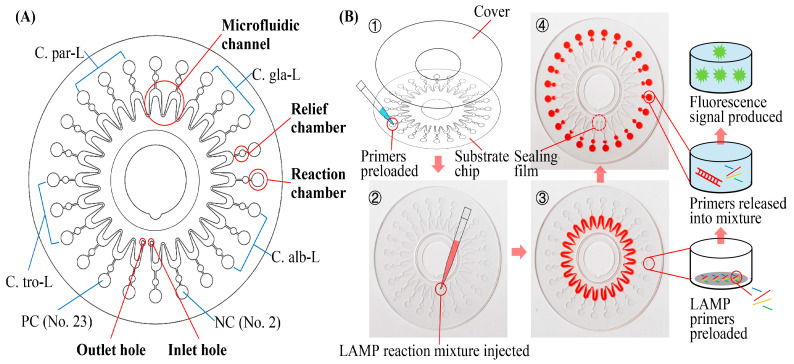
Schematic diagram, pictures and working principle of the microfluidic chip. (**A**) Structure of the microfluidic chip. (**B**) Pictures and working principle of the microfluidic chip. C.alb-L, C.gla-L, C.par-L, and C.tro-L represent the LAMP primer sets preloaded into the corresponding reaction chambers for the detection of *C. albicans*, *C. glabrata*, *C. parapsilosis*, and *C. tropicalis*, respectively. The LAMP reaction mixture was transparent; red pigment was added into the mixture to display the position of the mixture in the chip pictured. PC, positive control; NC, negative control; No., number. LAMP primers detecting 18S rRNA gene of *Plasmodium falciparum* and plasmids containing the corresponding sequences were preloaded in the chamber PC.

The disk-type microfluidic chip used in this work is shown in Figure 2. The chip consists of the substrate chip with microfluidic channels and a cover with double-faced adhesive tape (Figure 2B). The chips were fabricated using polycarbonate. The diameter and thickness of the chips were 60 mm and 0.6 mm, respectively. There were 24 reaction chambers uniformly distributed around the chip, each with a diameter and depth of 3 mm and 0.2 mm, respectively, and a volume of approximately 1.41 µL. Each reaction chamber was preloaded with one type of LAMP primer set in a fixed order (No. 2 for negative control, No. 4–6 for *C. albicans*, No. 9–11 for *C. glabrata*, No. 14–16 for *C. parapsilosis*, No. 19–21 for *C. tropicalis*, and No. 23 for positive control, as shown in Figure 2A). The primers were mixed with low melting-point agarose (0.07%, *w*/*v*), and then spotted and dried at room temperature in the corresponding reaction chamber. Besides primers, other components used in LAMP reaction mixture would be added as hydration mix. Primers in different chambers were used for the detection of different targets. The detailed sequences of the primers used in the microfluidic chips are shown in Table S1. The LAMP reaction mixture was prepared in the reagent storage unit of the system. Next, the smart injector automatically transferred the mixture into the chip, using the specially designed needle of the injector. The mixture was injected into the chip through the inlet hole. Then, the chip was centrifuged at 4000 rpm for 30 s, resulting in the dispensing of the mixture into each separate chamber. The relief chambers were designed to guarantee the air escape from reaction chambers into microfluidic channels and the mixture pushed outwards easily. The channel between the relief and reaction chambers was wide (0.5 mm) and short (1 mm), making the air in reaction chambers easily escaped into the relief chambers. After that, the air was immediately pushed into the microfluidic channel by the reaction mixture passing through relief chambers. The preloaded primers were released into the chamber when the chip was heated. Then, the targets were amplified by the primers, followed by the production of fluorescence signal with the combination of a fluorescent dye and the amplicons.

### 2.3. Preparation of Mycological Typing LAMP Primers

All mycological typing LAMP primers were designed using PrimerExplorer V5 (http://primerexplorer.jp/lampv5e/index.html, accessed on 23 June 2021) based on the specific ITS (internally transcribed spacer) sequence of each *Candida* [25,31]. All primers were synthesized by Sangon Biotech (Shanghai, China) with PAGE purification. In order to obtain the LAMP primers with high sensitivity and specificity, the primers were screened carefully. Detailed information is presented in Section 3 of Supplementary Materials, including Figures S2–S8 and Table S2. PCR primers used in this study were selected from the corresponding LAMP primers. The sequences of the primers are shown in Table S1.

### 2.4. Preparation of Nucleic Acids and Clinical Samples

Standard strains of microorganisms were used for the validation of primers. *C. albicans* (ATCC 10231), *C. glabrata* (GDMCC 2.210), *C. parapsilosis* (CICC 1676), and *C. tropicalis* (CICC 1254) were purchased from ATCC (American Type Culture Collection), GDMCC (Guangdong Microbial Culture Collection Center), or CICC (China Center of Industrial Culture Collection). They were cultured in a liquid malt extract medium (Hopebio, China) at 30 °C for 24 h before the extraction of nucleic acids. The concentration of the cultured *Candida* was measured through the flat colony counting method and calculated using colony forming units (CFU). *Lactobacillus crispatus* (*L. crispatus*, CICC 24879), *Gardnerella vaginalis* (*G. vaginalis*, GDMCC 1.1347), *C. krusei* (CICC 1273), *Escherichia coli* (*E. coli*, CICC 10003), *Staphylococcus aureus* (*S. aureus*, CICC 10001), *Streptococcus agalactis* (*S. agalactis*, CICC 10465), *Pseudomonas aeruginosa* (*P. aeruginosa*, CICC 10351), and the HeLa cell line (ATCC CRM-CCL-2) were also used in this work. To extract genomic DNA from cultured microorganism and cells, we used a DNeasy Blood & Tissue Kit (Qiagen, Germany) according to the recommended protocol. The extracted DNA was analyzed using a Qubit 3.0 (Invitrogen, United State) and electrophoresis on 1% agarose gels. At the same time, the sample processing cassette processed the microorganisms harvested from 1 mL of cultured *Candida*, through centrifugation, to extract nucleic acids used for detection.

The clinical samples were provided by Beijing Tsinghua Changgung Hospital. Ethical approval had been approved by the Ethics Committee of the Beijing Tsinghua Changgung Hospital (19204-0-03). The lateral walls of the vagina at the upper 1/3 position were sampled with two swabs. Further, the two swabs were rubbed against each other to ensure there were no differences between the two swabs. One swab was used to make vaginal discharge smears for Gram staining and then exanimated by an experienced clinical scientist using a microscope (Nikon eclipse 80i, Japan). When the *Candida* budding yeasts, hyphae, or pseudohyphae were found under the microscope, the sample was diagnosed as VVC, with their absence leading to a diagnosis of non-VVC (when other symptoms were found, such as bacterial vaginosis (BV), aerobic vaginitis (AV), etc.) or normal (when no symptoms were found). Concurrently, the residue of the swab was used for the nucleic acid extraction with the commercial kit. The other swab was stored at −20 °C until processed and analyzed by the RPT system. 

### 2.5. LAMP and PCR Assays

The LAMP assay was conducted using a WarmStart LAMP Kit (NEB, United States) in a 10 µL reaction system. WarmStart LAMP Master mix (5 µL), 1 μL LAMP primer mix (10 µM), 3.8 μL template, and 0.2 μL fluorescent dye (50× LAMP Fluorescent Dye, provided in the Kit) were mixed. The LAMP reaction was initiated at 37 °C for 3 min, and then incubated at 65 °C for 40 min with fluorescence signal measured every minute. This LAMP Kit could accomplish LAMP and RT-LAMP (reverse transcription LAMP) at the same time at 65 °C, as the Kit contains reverse transcriptase WarmStart RTx (NEB, United States). The 37 °C step was designed to ensure the RT-LAMP could still occur when other LAMP reaction mixture was used, containing reverse transcriptase requiring lower temperature for reverse transcription, such as AMV reverse transcriptase. The same LAMP reaction system was also used in the microfluidic chip and analyzed by the RPT system, with the volume set at 35 µL. The PCR assay was conducted using Hemo KlenTaq (NEB, United States) in a 10 µL reaction system. Hemo KlenTaq Reaction Buffer (2 µL), 0.2 µL of dNTPs (10 µM), 0.1 µL of SYBR Green I (100×), 0.6 µL of primer mix (10 µM), 0.8 µL of Hemo Klen Taq, 2.5 µL of ddH_2_O, and 3.8 µL of template were mixed. The thermal cycling conditions were set as follows: initial denaturation at 95 °C for 3 min and 40 cycles of denaturation step at 95 °C for 30 s, annealing at 55 °C for 30 s, and extension at 68 °C for 1 min. The fluorescence signal was measured at the end of each cycle. In addition, the melt curve signal was also collected to distinguish primer dimers and amplified targets. Both assays were performed using an ABI 7500 (Applied Biosystems, Waltham, MA, USA).

## 3. Results and Discussion

### 3.1. Sensitivity of the LAMP Primers

**Figure 3:**
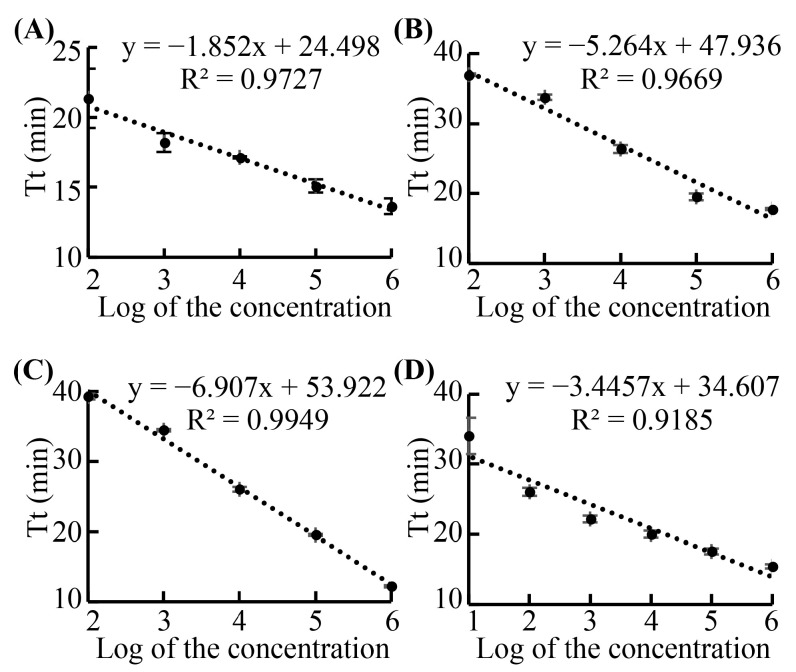
(**A**–**D**) represent the linearity analysis for *C. albicans*, *C. glabrata*, *C. parapsilosis*, and *C. tropicalis*, respectively. Tt, Threshold time; Log, logarithmic. Results were collected from three independent tests. Error bars indicate the standard deviation from the mean.

The sensitivity of the LAMP primer sets was characterized using the genomic DNAs extracted from the 10-fold serially diluted cultured *Candida* samples using commercial kits. For example, to test the sensitivity of the primer set for detecting *C. albicans*, genomic DNAs from *C. albicans* with different concentrations (from 2.23 × 10^6^ CFU/mL to 2.23 × 10^0^ CFU/mL) were used as templates. Deionized water was used as the template in negative control reactions. The primer set used in this assay was named ‘C.alb-L’. As shown in Figure 3A, the LAMP primers could detect the *C. albicans* at the concentration as low as 2.23 × 10^2^ CFU/mL with a good linearity (R^2^ = 0.9727). The good linear relationship between the lg (concentration of *C. albicans*) and the threshold time of the reaction indicates that the assay was quantitative. The sensitivity of the primer sets used for detecting *C. glabrata*, *C. parapsilosis*, and *C. tropicalis*, named ‘C.gla-L’, ‘C.par-L’, and ‘C.tro-L’, was also characterized. The sensitivities of the ‘C.gla-L’, ‘C.par-L’, and ‘C.tro-L’ sets were 8.10 × 10^2^ CFU/mL, 3.53 × 10^2^ CFU/mL, and 2.80 × 10^1^ CFU/mL, respectively, with good linearity as well (Figure 3B–D). As the volume of the template used in each LAMP assay was set at 3.8 µL, the detection sensitivities of the LAMP primers using commercial kits and LAMP assays were 0.85 CFU/reaction, 3.08 CFU/reaction, 1.34 CFU/reaction, and 0.11 CFU/reaction for *C. albicans*, *C. glabrata*, *C. parapsilosis*, and *C. tropicalis*, respectively.

### 3.2. Performance of the Sample Processing Cassette

Nucleic acid extraction efficiency of the sample processing cassette of the RPT system and commercial kit were compared through LAMP and PCR assays. For example, various dilutions of the cultured *C. tropicalis* (from 2.80 × 10^6^ CFU/mL to 2.80 × 10^0^ CFU/mL) were simultaneously processed by the cassette and kit. Subsequently, the extracted nucleic acids were detected by LAMP and PCR assays. Table 1 summarizes the detection sensitivity and linearity of the LAMP and PCR assays for detecting *Candida* samples processed through two different methods. The detailed results are shown in Figure 3 and Figures S9–S11. For the LAMP assay, the sensitivity of detecting *C. albicans* and *C. tropicalis* processed by the cassette was equal to that processed by the kit. As for *C. glabrata* and *C. parapsilosis*, the detection sensitivity using a cassette was one order of magnitude higher than that using the kit. For PCR assay, a similar pattern was found, except that the detection sensitivity of *C. tropicalis* processed by the cassette was also higher than that processed by the kit. These results demonstrate that in terms of detection sensitivity, the nucleic acids extracted by the sample processing cassette could be detected as well as that processed by the commercial kit. In addition, the sensitivity of the LAMP assay was equal to that of PCR assay or only within one order of magnitude. The high sensitivity of the LAMP assays, equal to or near the corresponding PCR assays, was also related to the good performance of the cassette. At the grinding step, the heating and vibration unit could vibrate the smart injector not only horizontally, but also vertically, as shown in Video S1. The thorough vibration of the injector and grinding using glass beads guaranteed the efficiency of the cell lysing and nucleic acids releasing.

### 3.3. Detection Sensitivity and Specificity of the RPT System

**Figure 4:**
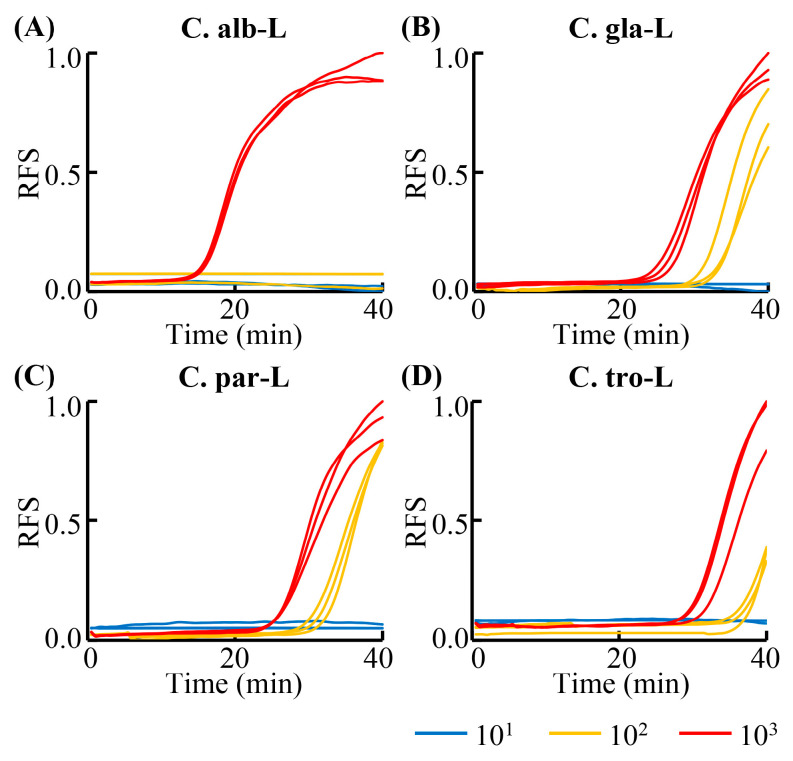
Detection sensitivity results of RPT system assays. (**A**–**D**) represent the detection sensitivity results of RPT system assay using the LAMP primer set C.alb-L, C.gla-L, C.par-L, and C.tro-L, respectively. C.alb-L, C.gla-L, C.par-L, and C.tro-L represent the LAMP primers sets used for the detection of *C. albicans*, *C. glabrata*, *C. parapsilosis*, and *C. tropicalis*, respectively. The coefficients of the concentrations of the *C. albicans*, *C. glabrata*, *C. parapsilosis*, and *C. tropicalis*, were 2.75, 4.03, 3.48, and 3.31, respectively. 10^1^–10^3^ represents the concentration of the sample used; RFS, relative fluorescent signal. Results were collected from three independent tests.

As the volume of the reaction mixture in each reaction chamber of the chip was set at 1.41 µL, lower than that of 10 µL LAMP assay, the detection sensitivity of the RPT system was verified. For example, various dilutions of the cultured *C. tropicalis* at low concentrations (from 3.31 × 10^3^ CFU/mL to 3.31 × 10^1^ CFU/mL) were processed and detected by the RPT system. As shown in Figure 4D, the LAMP primers could detect the *C. tropicalis* at the concentration as low as 3.31 × 10^2^ CFU/mL. The sensitivities of detecting *C. albicans*, *C. glabrata*, and *C. parapsilosis* using the RPT system were 2.75 × 10^3^ CFU/mL, 4.03 × 10^2^ CFU/mL, and 3.48 × 10^2^ CFU/mL. The detection sensitivity of the concentration of the sample using RPT system was lower than that of LAMP assays using the sample processing cassette, because of the volume of the reaction mixture decreased. However, as the volume of the reaction mixture used in each reaction chamber was set at 1.41 µL, the detection sensitivities of the LAMP primers using the RPT system were 1.47 CFU/reaction, 0.22 CFU/reaction, 0.19 CFU/reaction, and 0.18 CFU/reaction for *C. albicans*, *C. glabrata*, *C. parapsilosis*, and *C. tropicalis*, respectively, indicating the detection sensitivity maintained in each reaction. The detection sensitivities were approximately or even lower than 1 CFU/reaction. In addition to the fact that the 1 CFU normally represents greater than one copy of DNA, the good performance of the RPT system may be guaranteed from two other aspects. Firstly, the nucleic acid-releasing efficiency of the sample processing cassette was high, as discussed earlier. Secondly, presumably not only the genomic DNAs, but the RNAs of the cells were also preserved and used for detection in the RPT system, as the process is rapid, and the heating could deactivate RNases [39]. Meanwhile, the WarmStart LAMP Kit used in this work could not only amplify DNAs, but also RNAs, as the enzymes used for revers transcription added into the LAMP Kit [39]. In practice, the positive reaction signals could be identified within 30 min when the concentration of the targets were not too low. Considering the shorter reaction time (<1 h for LAMP vs. >2 h for PCR), the RPT system would also have excellent performance in sensitivity and efficiency in clinical sample assays [38].

**Figure 5:**
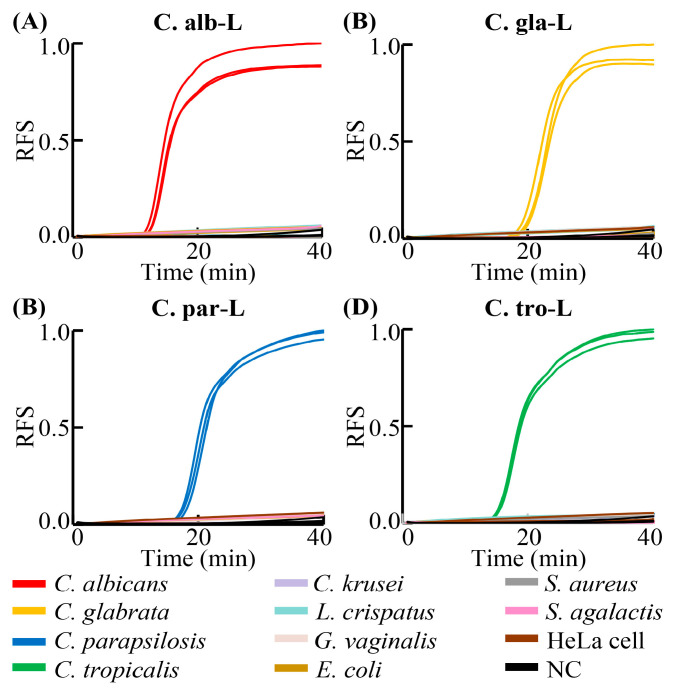
Detection specificity results of RPT system assays. (**A**–**D**) represent the detection specificity results of RPT system assay using the LAMP primer set C.alb-L, C.gla-L, C.par-L, and C.tro-L, respectively. C.alb-L, C.gla-L, C.par-L, and C.tro-L represent the LAMP primers sets used for the detection of *C. albicans*, *C. glabrata*, *C. parapsilosis*, and *C. tropicalis*, respectively. RFS, relative fluorescent signal; NC, negative control. Results were collected from three independent tests.

In addition, considering that there may not be only *Candida* species in clinical samples, the specificity of the RPT system needed to be characterized [3,4]. Here, we used five types of *Candida* species (*C. albicans*, *C. glabrata*, *C. parapsilosis*, *C. tropicalis*, and *C. krusei*) and six other types of cells that may be present in clinical samples (*L. crispatus*, *G. vaginalis*, *E. coli*, *S. aureus*, *S. agalactis*, and HeLa cells) to characterize the specificity of the RPT system [18,40,41]. Cultured samples at the concentration of 10^6^ CFU/mL (for *Candida* species and bacteria) or 10^6^ cells/mL (for HeLa cells) were used as samples in each RPT system assay. The results are shown in Figure 5. For example, when *C. albicans* was analyzed using C.alb-L, the amplified signal could be recorded by the system, meaning the detection result was positive. However, when other types of samples were used, the detection results were negative, with no amplification signal. As shown in Figure 5, all the RPT system assays using the four LAMP primer sets could detect and identify the *Candida* species with high specificity, meaning the assay could be used for the detection of *Candida* species and diagnosis of VVC for clinical samples.

There have been many microfluidic systems or methods designed for nucleic acid detection [42,43]. Here, we compared the RPT system with relating works, especially detecting *Candida* species using LAMP. The full information is presented in Table 2. Most of the relating works utilized commercial kits for the extraction of nucleic acids, which were time consuming (normally >30 min) when compared with the rapid sample processing cassette in the RPT system. The rapid DNA extraction method using Chelex-100 could help finishing the whole assay in one hour [36]. However, the sensitivity of the assay decreased when compared with commercial kit used as control, and the cassette used in this work [36]. In addition, the sensitivity and specificity of the RPT system were high when compared with these relating works, as only 1.41 µL of LAMP reaction mixture being needed for each reaction decreased the cost.

### 3.4. Validation of the RPT System Assay with Clinical Samples

Clinical samples from 32 patients (ages 18–63) were processed and analyzed by the RPT system, with the extracted nucleic acids from kits analyzed by PCR assays. The results are summarized in Table 3, and the detailed results are shown in Tables S3 and S4. Among the 32 samples, 18 samples were diagnosed as VVC, 7 samples were diagnosed as non-VVC (AV or BV), and the remaining seven samples were normal. *Candida* species were found by LAMP assays in all 18 VVC samples. One *Candida* species was detected in each sample. This is rational, because >90% of VVC samples contain only one *Candida* species [1]. Briefly, *C. albicans*, *C. glabrata*, *C. parapsilosis*, and *C. tropicalis* were found in nine, five, one, and three clinical samples, respectively. At the same time, there were no *Candida* species found in non-VVC and normal swab samples, representing the specificity of the RPT system assay was confirmed again. The clinical sensitivity and specificity of RPT system assay were both 100% for the analyzed clinical samples in this work, indicating that the RPT system could be used for the analysis of clinical samples (Table 4). Furthermore, as the detection results were consistent with that of the PCR assays, the high consistency between the RPT system assay and PCR assay on the ABI7500 machine indicates the effectiveness of the RPT system.

The identification of the *Candida* species for the non-*albicans* infections is important in clinical practice. For example, the symptoms are milder with *C. glabrata*, but it is difficult to recognize *C. glabrata* by microscope [3,44]. At this point, the identification of *C. glabrata* using the RPT system is useful and essential for the diagnosis of VVC. In the future, to fully test the performance of the RPT system, more clinical samples need to be collected and tested. Further, it would be better if the culture results, detection results using commercial clinical kits, and sequencing results could be collected at the same time [28]. With the participation of a microfluidic chip, the multiplex identification of the *Candida* species was realized in a reaction. Each reaction chamber only used 1.41 µL of reaction mixture, which was low-cost when compared to the reaction conducted in PCR tubes (at least 10 µL for each tube). As there were 24 reaction chambers in a chip, up to 22 targets (besides negative and positive controls) could be detected at the same time in theory. Therefore, more LAMP primer sets could be designed and screened to detect additional *Candida* species related to VVC, such as *C. krusei*, or to identify the genes or mutations related to the drug resistance of *Candida* species [1,45,46,47]. This would aid in the precise diagnosis and personalized treatment of VVC.

### 3.5. Period of Validity of the Reagents and Chips Used in the RPT System

**Figure 6:**
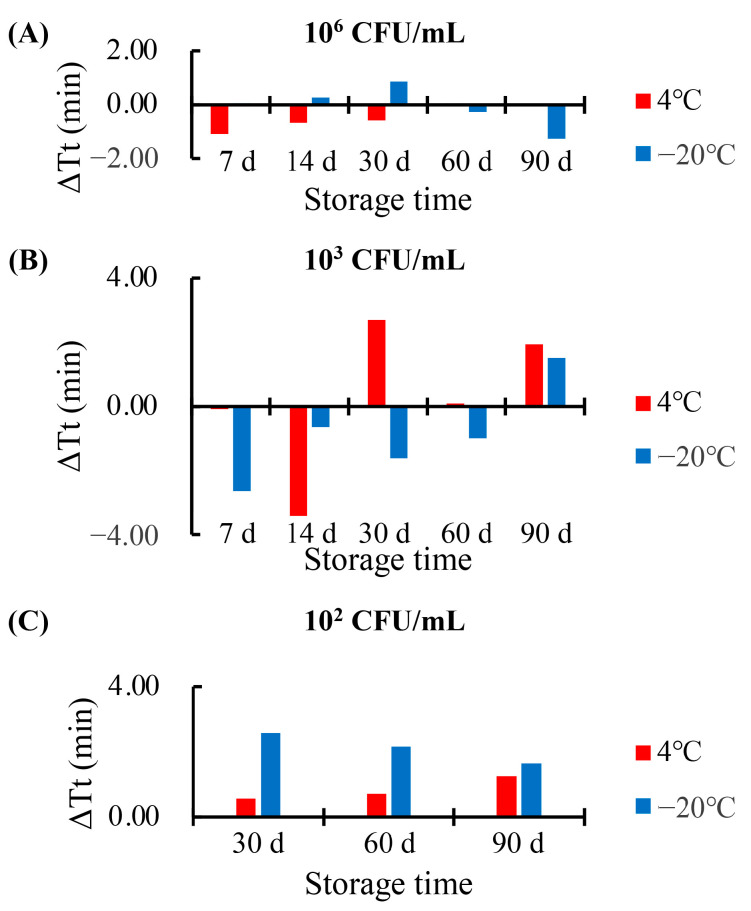
Detection results using pre-prepared reagent and chips after a period of storage. (**A**) Detection results of *C. tropicalis* at the concentration of 10^6^ CFU/mL. (**B**) Detection results of *C. tropicalis* at the concentration of 10^3^ CFU/mL. (**C**) Detection results of *C. tropicalis* at the concentration of 10^2^ CFU/mL. CFU, colony forming units; Tt, threshold time. Mean. Results were collected from three independent assays and are shown as the difference between the mean values.

As there may be no laboratory conditions in resource-limited regions for freshly preparing reagents before use, the period of validity of the reagents used in the RPT system was validated. The lysis buffer, LAMP reaction reagents, and chips were prepared uniformly. The LAMP reaction reagents were stored at −20 °C. The lysis buffer and chips were stored at 4 °C or −20 °C. After a period of 7 days, 14 days, 30 days, 60 days, and 90 days, the stored reagents and chips were used to detect the *C. tropicalis* at different concentrations. At the same time, freshly prepared reagents and chips were also used as controls. The difference value of the threshold time (Tt) between the pre-prepared and freshly prepared reagents and chips was calculated, as shown in Figure 6. When the difference of Tt (Δ*Tt*) is a positive value, the Tt of pre-prepared reagents and chips is higher than that of fresh prepared reagents, and vice versa. When the concentration of the sample was high (at 10^6^ CFU/mL), the numerical value of the Δ*Tt* was lower than 1.5 min (Figure 6A), indicating the effectiveness of the pre-prepared reagents and chips after a storage time of 90 days. When the concentration of the sample was low (at 10^3^ CFU/mL), the numerical value of the Δ*Tt* increased, indicating that the stability of the pre-prepared reagents and chips may change slightly after a long storage period. However, the effectiveness of the stored reagents and chips was still observed (Figure 6B). A similar pattern was also observed when the concentration was lower (at 10^2^ CFU/mL, near the limit of the detection of C.tro-L) in Figure 6C, and this result proved the detection sensitivity of the RPT system assay could be maintained after the 90 days storage. Furthermore, the difference of the lysis buffer and chips stored at different temperature was not significant in this test, as the differences of Δ*Tt* between the two temperatures were lower than 3 min. Hence, the period of validity of the reagents and chips used in the RPT system was at least 90 days, indicating its feasibility of application in resource-limited regions.

### 3.6. Processing and Detecting Ability of Bacteria Using the RPT System

**Figure 7:**
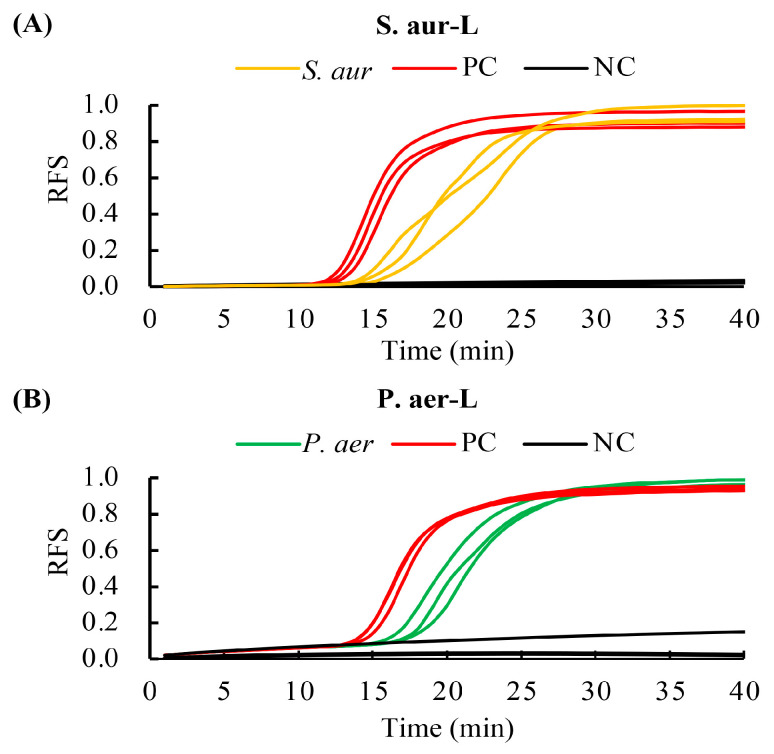
Detection results of bacteria processed by the RPT system. (**A**) Detection results of *S. aureus* after processing by the cassette. (**B**) Detection results of *P. aeruginosa* after processing by the cassette. The coefficients of the concentrations of the *S. aureus* and *P. aeruginosa* were 5.77 and 3.63, respectively. S.aur-L and P.aer-L represent the LAMP primer sets used for the detection of *S. aureus* and *P. aeruginosa*. *S. aur*, *S. aureus*; *P. aer*, *P. aeruginosa*; PC, positive control; NC, negative control; and RFS, relative fluorescent signal. Results were collected from three independent tests.

The ability of the RPT system to process and detect bacteria was also validated. The lysis buffer in the cassette was the same as previously described, except for an additional 10 mg lysozyme (Solarbio, China) [48]. Cultured *S. aureus* (Gram-positive) and *P. aeruginosa* (Gram-negative) at a concentration of 10^4^ CFU/mL were processed by the cassette and detected by the microfluidic chips using LAMP assays. Genomic DNAs of the two bacteria were used as templates in positive control reactions, with deionized water used as a negative control. As shown in Figure 7, *S. aureus* and *P. aeruginosa* could still be successfully processed by the cassette and detected by LAMP assays, especially the Gram-positive bacteria.

## 4. Conclusions

In this work, an integrated and multi-target nucleic acid analysis system, called the RPT system, used for the rapid and isothermal identification of four *Candida* species was proposed. The system could identify *Candida* species with high sensitivity (<2 CFU/reaction) and high specificity. The sample processing cassette of the RPT system could process samples rapidly (15 min) and automatically. The detection sensitivity of the processed samples was equal to or even higher than that using commercial kits. In addition, the specificity of the RPT system assay was also high. The whole processing and analyzing procedures could be finished within 60 min by the RPT system, which is much shorter than that using commercial kits and PCR assays (>4 h). These make the RPT system an effective tool in the aera of rapid detection of *Candida* species. Clinical samples were also processed and analyzed with the RPT system. The results demonstrated high clinical sensitivity and specificity, indicating the effectiveness of the RPT system for the diagnosis of VVC. As the period of validity of the reagents and chips used in the RPT system was proved to be at least 90 days, it is promising to use the RPT system in the resource limited regions with pre-prepared reagents and chips. In addition, the feasibility of bacteria detection using the RPT system was also validated in this work, expanding the analysis range of the targets using the system.

## Figures and Tables

**Table 1:** The detection sensitivity of LAMP and PCR assays.

**Species**		***C. albicans***		***C. glabrata***	
**Method**		**SPC**	**Kit**	**SPC**	**Kit**
LAMP	DS (CFU/mL)	2.23 × 10^2^	2.23 × 10^2^	8.10 × 10^1^	8.10 × 10^2^
	R^2^	0.9795	0.9727	0.9754	0.9669
PCR	DS (CFU/mL)	2.23 × 10^1^	2.23 × 10^1^	8.10 × 10^1^	8.10 × 10^2^
	R^2^	0.9894	0.9953	0.9569	0.9991
**Species**		***C. parapsilosis***		***C. tropicalis***	
**Method**		**SPC**	**Kit**	**SPC**	**Kit**
LAMP	DS (CFU/mL)	3.53 × 10^1^	3.53 × 10^2^	2.80 × 10^1^	2.80 × 10^1^
	R^2^	0.9405	0.9949	0.9690	0.9185
PCR	DS (CFU/mL)	3.53 × 10^0^	3.53 × 10^1^	2.80 × 10^0^	2.80 × 10^1^
	R^2^	0.9869	0.9992	0.9909	0.9894

SPC: sample processing cassette, representing the sample processing cassette used; Kit: commercial kit, representing the commercial kit used; DS, detection sensitivity; CFU, colony forming units. Results were collected from three independent tests.

**Table 2:** Comparation of microfluidic systems detecting *Candida* species using LAMP.

System/Method	Target	Sample	Sensitivity	Specificity	Cost	Efficiency	Ref
Membrane microarray hybridized with DIG-labeled LAMP amplicons	7 *Candida* species and 2 other yeasts	Cultured sample	>6 cells/reaction	High when detecting another 8 nontarget yeasts	~3.8 euros	~5–6 h for one assay	[30]
LAMP in PCR tubes	Including 4 *candida* species	Plasmids, swabs from indoor environment	≥10 plasmids/reaction	Universal primers for fungal species	N/A	~2–2.5 h for one assay	[32]
LAMP in PCR tubes	*Candida auris*	Cultured sample, ear swab	20 copies/reaction	High when detecting another 38 fungal species	25 µL LAMP mix per reaction	~2 h for one assay	[35]
PMAxx-LAMP with portable system	*Candida albicans*	Cultured sample	10^3^ CFU/mL	Distinguishing viable and dead cells	~1 µL LAMP mix per reaction	~2 h for one assay	[29]
Microfluidic chip and detector (iChip-400, Baicare) using LAMP	*Candida albicans* and 4 other species	Cultured sample, clinical sample	463 pg/µL	8 negative and 2 false positive results for another 10 species	~7 µL LAMP mix per reaction	~1.5 h or one assay	[33]
LAMP in PCR tubes, combined with rapid DNA extraction using Chelex-100	Universal primers for 6 *candida* species	Cultured sample, blood	>10^4^ cells/mL	High when detecting another 5 species	25 µL LAMP mix per reaction	~1 h for one assay	[36]
Microfluidic chip and detector (iChip-400, Baicare) using LAMP	*Candida albicans* and 4 other species	Cultured sample, clinical sample	7.53 CFU/µL	High when detecting another 10 species	~7 µL LAMP mix per reaction	~1.5 h or one assay	[34]
The RPT system	4 *candida* species	Cultured sample, Vaginal swab	<2 CFU/reaction	High when detecting corresponding another 10 species	1.41 µL LAMP mix per reaction	<1 h for one assay	This work

DIG, digoxigenin; N/A, not applicable; PMAxx, propidium monoazide; CFU, colony forming unit. One assay consists of sample processing, DNA extraction, and LAMP detection. The time used for DNA extraction using commercial kit was estimated as 30 min in relating works, though it may be longer in fact.

**Table 3:** Detection results of clinical samples using LAMP and PCR assays.

Clinical Samples		Species	LAMP	PCR
VVC	18	*C. albicans*	9	9
		*C. glabrata*	5	5
		*C. parapsilosis*	1	1
		*C. tropicalis*	3	3
non-VVC	7	No *Candida* species were detected.		
Normal	7			

‘LAMP’ represents the LAMP assay using the RPT system; ‘PCR’ represents the PCR assay using the ABI7500 machine. Results were collected from three independent assays.

**Table 4:** Authenticity and consistency evaluation of LAMP and PCR assays.

Methods	Microscopic Examination		Sensitivity	Specificity	Positive Predictive Value	Negative Predictive Value	Kappa Value
	Positive	Negative					
LAMP			100%	100%	100%	100%	1
Positive	18	0					
Negative	0	14					
PCR			100%	100%	100%	100%	1
Positive	18	0					
Negative	0	14					

‘LAMP’ represents the LAMP assay using the RPT system; ‘PCR’ represents the PCR assay using the ABI7500 machine; ‘Positive’ represents the ‘VVC’ diagnostic results or *Candida* species detected; ‘Negative’ represents the ‘non-VVC’ and ‘Normal’ diagnostic results or *Candida* species not detected. Results were collected from three independent assays.

## Data Availability

Data is contained within the article or supplementary material.

## Supplementary Materials

The following supporting information can be downloaded at: https://www.mdpi.com/article/10.3390/bios13050559/s1, Figure S1: Images of the system and smart injector. (A) Images of the system. (B) Images of the smart injector at the state of aspirating and dispensing reagents; Figure S2: Structure and cross section of the fluorescent signal detector; Figure S3: ITS sequence alignment of *C. tropicalis*, *C. albicans*, *C. glabrata*, *C. parapsilosis*, and *Candida krusei* (GenBank no. NC_042506.1); Figure S4: Selection of specificity of C.tro-L1 to C.tro-L9; Figure S5: Selection of sensitivity of C.tro-L2 and C.tro-L7; Figure S6: Verification of sensitivity of C.tro-L2 and C.tro-L7; Figure S7: Verification of the secondary structure of C.tro-L2 and C.tro-L7; Figure S8: Verification of specificity of C.tro-L2 and C.tro-L7; Figure S9: Detection sensitivity and linearity analysis of *Candida* species using the sample processing cassette and LAMP assays; Figure S10: Detection sensitivity and linearity analysis of *Candida* species using a commercial kit and PCR assays; Figure S11: Detection sensitivity and linearity analysis of *Candida* species using the sample processing cassette and PCR assays; Table S1: Sequences of the primers used for LAMP and PCR assays in this work; Table S2: Screening results of each step of the ‘five-step screening’ method; Table S3: Diagnosis results and detection results of clinical samples; Table S4. Original detection results of clinical samples; Video S1: Vibration of smart injector.
