# Supplementary material for: An Integrated and Multi-Target Nucleic Acid Isothermal Analysis System for Rapid Diagnosis of Vulvovaginal Candidiasis
Source: Biosensors (Basel). 2023 May 19;13(5):559. doi: 10.3390/bios13050559 (PMC10216317; doi:10.3390/bios13050559)
Supplement: Supplementary file 1 [file biosensors-13-00559-s001.zip › biosensors-2341421-supplementary.pdf]

Supplementary Materials

An Integrated and Multi-Target Nucleic Acid Isothermal Analysis System for Rapid Diagnosis of Vulvovaginal Candidiasis

Xiangyu Jin [1,†], Meng Li [2,†], Zeyin Mao [1], Anni Deng [1], Wenqi Lv [1], Leyang Huang [1], Hao Zhong [1], Han Yang [1], Lei Zhang [2], Qinping Liao [2,] and Guoliang Huang [1,3,*]

[1] Department of Biomedical Engineering, School of Medicine, Tsinghua University, Beijing 100084, China
[2] Department of Obstetrics and Gynecology, Beijing Tsinghua Changgung Hospital, School of Clinical Medicine, Tsinghua University, Beijing 102218, China; zla00969@btch.edu.cn (L.Z.); lqpa00594@btch.edu.cn (Q.L.)
[3] National Engineering Research Center for Beijing Biochip Technology, Beijing 102206, China
* Correspondence: tshgl@mail.tsinghua.edu.cn
† These authors contributed equally to this work.

Table of contents

Section 1: Images of the system 2
Section 2: Sequences of primers used in this work 4
Section 3: Detailed information of lamp primer design and screening 5
Section 4: Validation of the performance of the sample-processing cassette 11
Section 5: Clinical sample results 13

Citation: Jin, X.; Li, M.; Mao, Z.; Deng, A.; Lv, W.; Huang, L.; Zhong, H.; Yang, H.; Zhang, L.; Liao, Q.; et al. An Integrated and Multi-Target Nucleic Acid Isothermal Analysis System for Rapid Diagnosis of Vulvovaginal Candidiasis. *Biosensors* **2023**, *13*, x. https://doi.org/10.3390/xxxxx

Received: 6 April 2023
Revised: 9 May 2023
Accepted: 16 May 2023
Published: 19 May 2023

Copyright: © 2023 by the authors. Submitted for possible open access publication under the terms and conditions of the Creative Commons Attribution (CC BY) license (https://creativecommons.org/licenses/by/4.0/).

Section 1: Images of the system

The images of the system and smart injector are shown in Figure S1. Figure S1A shows a real image of the whole system, including the sample-processing cassette, the microfluid chip, and the fluorescent signal detector. In the real system, the microfluidic chip was wrapped by heating panels from both sides. The heating panel of the upper side was removable for the convenience of loading the microfluidic chip and reagent storage unit. The microfluidic chip was controlled by the stepper motor through chain gearing. In the sample-processing cassette, the liquid control unit could control the plunger of the smart injector for aspirating and dispensing reagents during various operational steps, as shown in Figure S1B. The smart injector was also heated and vibrated by the heating and vibration unit. A video of the step is provided in Video S1.

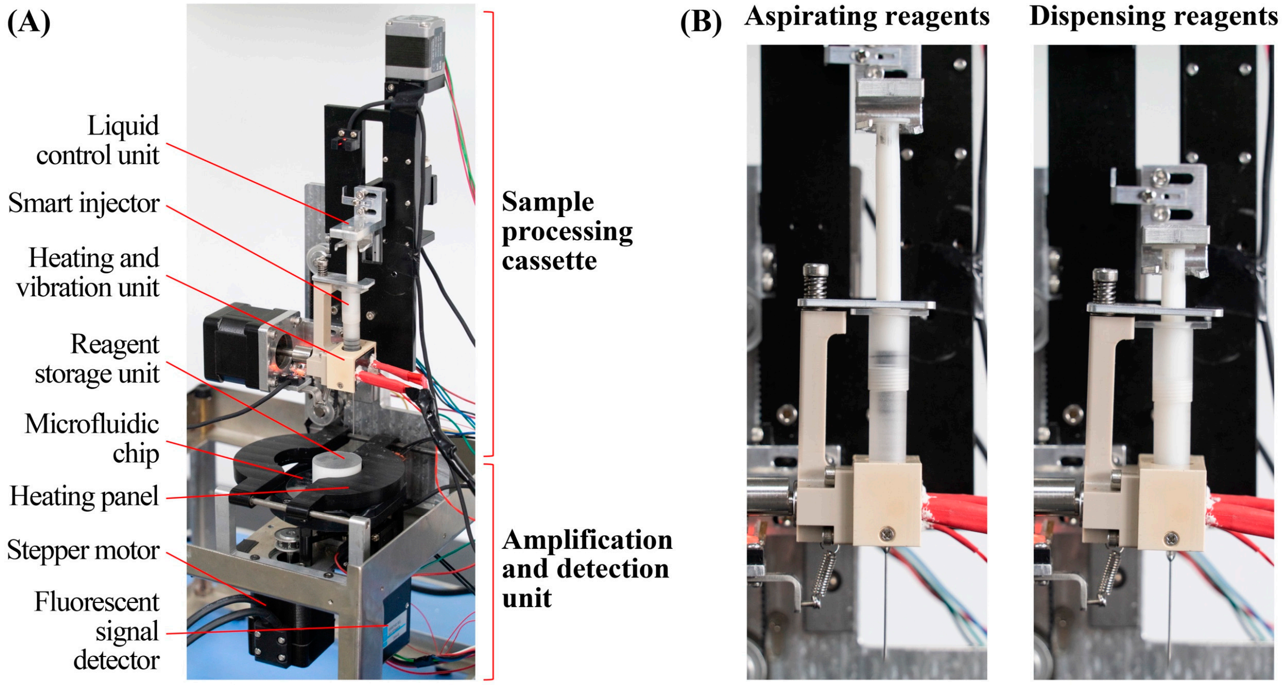

Figure S1. Images of the system and smart injector. (A) Images of the system. (B) Images of the smart injector at the state of aspirating and dispensing reagents.

In the amplification and detection unit, the fluorescent signal of the reaction chambers of the microfluidic chip was collected by a fluorescent signal detector. The detector consists of light-emitting diodes (LEDs), a lens battery, filters, and photomultiplier tube (PMT), as shown in Figure S2. The blue light (central wavelength = 470 nm) was emitted by an LED and was focused on the reaction chambers of the microfluidic chip, through the collecting lens, the excitation filter (central transmittance wavelength = 470 nm), the beam splitter, and objective lens in sequence. The fluorescent dye was excited and emitted green light (central wavelength = 520 nm) when combined with amplicons. Then, the green light was collected via the objective lens and reflected to the PMT by the beam splitter, through the emission filter (central transmittance wavelength = 520 nm), imaging lens, and aperture stop in sequence. The PMT transformed the optical signal into an electrical signal, which was later transmitted and eventually recorded by a computer.

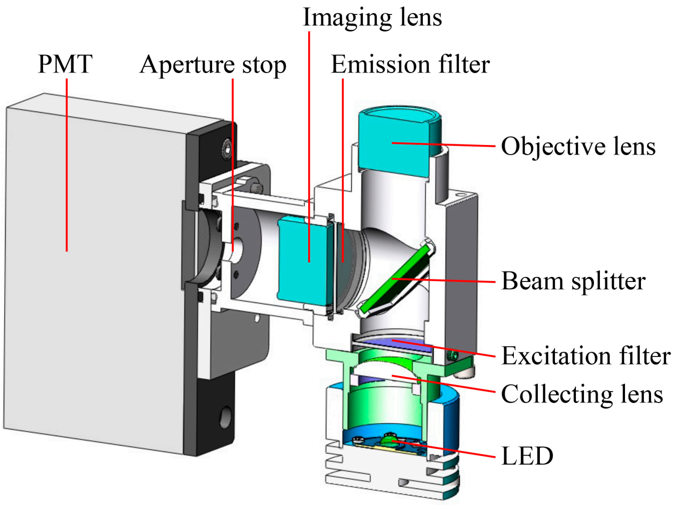

Figure S2. Structure and cross section of the fluorescent signal detector.

Section 2: Sequences of primers used in this work

Table S1. Sequences of the primers used for LAMP and PCR assays in this work

Name	Sequence		Target
C.alb-L	F3	GTTGGCAAAAATATCAATGGTAA	*Candida albicans*
	B3	GTAAACTCAGAAGCTGGAAC	
	FIP	AGCTTGGAAGGCATCAATAATATCTGTGTGTATCGATGTTCTTTTAGATTCTGGT	
	BIP	GACGGTCAAGGTCATACTTTCTAGTGTGTTGAAATCTTGGCGTTGTTGTC	
	LF	TGAGCAACATCTTGTTGAAGATAAGT	
	LB	TTACTGATTGTCAAACTTCTGGAACT	
C.gla-L	F3	TGGTAGTGAGTGATACTCGTT	*Candida glabrata*
	B3	CTTAAAGACGTCTGTCTGCC	
	FIP	GCAGATTAATAGAGAAGCTTGCGCTGAGTTAACTTGAAATTGTAGGCC	
	BIP	GCGGCGGGGGTTAATACTGTCACAAAACACTCACTTATCCCT	
	LF	GGTTTTACCAACTCGGTGTTGATCT	
C.par-L	F3	GCGATAAGTAATATGAATTGCAGAT	*Candida parapsilosis*
	B3	GAAGTTTTGGAGTTTGTACCA	
	FIP	AGGCATGCCCTTTGGAATACCTGTGTGATTCGTGAATCATCGAATCTTTG	
	BIP	GTCATTTCTCCCTCAAACCCTCGTGTGTGCATTAGTTTATACTCCGCCTTTC	
	LB	GTGTTGAGCGATACGCTGGG	
C.tro-L	F3	GTTTGAGCGTCATTTCTCC	*Candida tropicalis*
	B3	CTGTTGGTTTCTTTTCCTCC	
	FIP	GTCGCTTAAAATAAGTTTCCACGTTTGTGTGGGGTTTGGTGTTGAGCAATA	
	BIP	TTGCTAGTGGCCACCACAATTGTGTGGCTTAAGTTCAGCGGGTAG	
	LB	AACTTTGACCTCAAATCAGGTAGG	
S.aur-L	F3	GTGCCTTTACAGATAGCATG	*Staphylococcus aureus*
	B3	GAAAAAGTGTACGAGTTCTTGA	
	FIP	GTTTCATAACCTTCAGCAAGCTTTTGTGTGCCATACAGTCATTTCACGCA	
	BIP	GAGGTCATTGCAGCTTGCTTACTTTGTGTGCGATCACTGGACCGCG	
	LF	AACTCATAGTGGCCAACA	
	LB	GTACCTGTTATGAAAGTGTTCA	
P.aer-L	F3	TGTTATGGAAATGTCCACCTT	*Pseudomonas aeruginosa*
	B3	TCTGCTGAGCTTTCTGAG	
	FIP	GGCCAGAACAGCAGCCAGAGGAACACGATGAACAACGT	
	BIP	AGCTCGTCTGACCGCTACCCTTCGTCAGCCTTGCGAT	
	LF	CCAGAGCAGAGAATTTCAGA	
	LB	AGCTGCTCGTGCTCAGG	
C.alb-P	FP	GTTGGCAAAAATATCAATGGTAA	*Candida albicans*
	RP	GTAAACTCAGAAGCTGGAAC	
C.gla-P	FP	TGGTAGTGAGTGATACTCGTT	*Candida glabrata*
	RP	CTTAAAGACGTCTGTCTGCC	
C.par-P	FP	GCGATAAGTAATATGAATTGCAGAT	*Candida parapsilosis*
	RP	GAAGTTTTGGAGTTTGTACCA	
C.tro-P	FP	AACGTGGAAACTTATTTTAAGCGAC	*Candida tropicalis*
	RP	CTGTTGGTTTCTTTTCCTCC	

Section 3: Detailed information of LAMP primer design and screening

Primer design based on ITS sequence. To design species-specific primers for each target, sequences of the internal transcribed spacer (ITS) of *Candida albicans* (GenBank no. NC_032096.1), *Candida glabrata* (GenBank no. NC_006035.2), *Candida parapsilosis* (GenBank no. NW_023503284.1), and *Candida tropicalis* (GenBank no. CP047875.1) were obtained from the NCBI GenBank database (https://www.ncbi.nlm.nih.gov/genbank/) and were aligned with sequences of other species of the same genus using the software SnapGene (Figure S3).

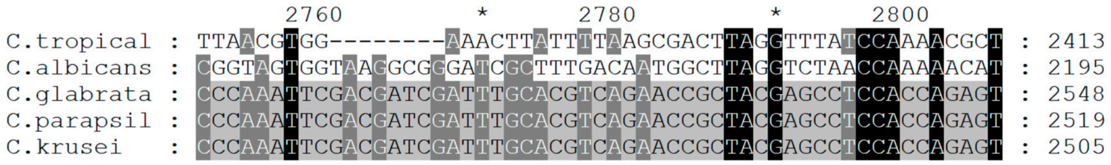

Figure S3. ITS sequence alignment of *C. tropicalis*, *C. albicans*, *C. glabrata*, *C. parapsilosis*, and *Candida krusei* (GenBank no. NC_042506.1). Bases that matched in at least three sequences are marked with different shades of highlighting according to the matching degree; the higher matching degree, the deeper the shade of highlighting. The long-enough segment with multiple unmatched bases (bases without shade of highlighting) was considered to be species-specific and suitable for primer design.

The primers were designed with PrimerExplorer V5 (http://primerexplorer.jp/lampv5e/index.html), using the species-specific segments from the alignment, according to the instructions of the software. To ensure the performance of the primers designed, each set of primers was checked using NCBI BLAST (https://blast.ncbi.nlm.nih.gov/Blast.cgi) to verify its specificity. UNAFold (http://www.unafold.org/) was used to examine the secondary structure of the primers, at the theoretical level. Finally, for each target, 9-12 sets of primers were synthesized by Sangon Biotech (Shanghai, China) and then became candidates in the following experimental primer selection.

Primer screening using the 'five-step screening' method. The process of primer selection included five steps, called 'five-step screening': selection of specificity, selection of sensitivity, verification of sensitivity, verification of secondary structure, and verification of specificity (Table S2). The screening of *C. tropicalis* primers is used as an example to introduce the whole process:

Step 1: Selection of specificity. To select primers that did not easily produce artefacts, LAMP reaction was performed in a 10 μL volume without template at 65°C for 60 min. In eight independent replicate runs, primers that yielded negative results were selected for the next step (Figure S4).

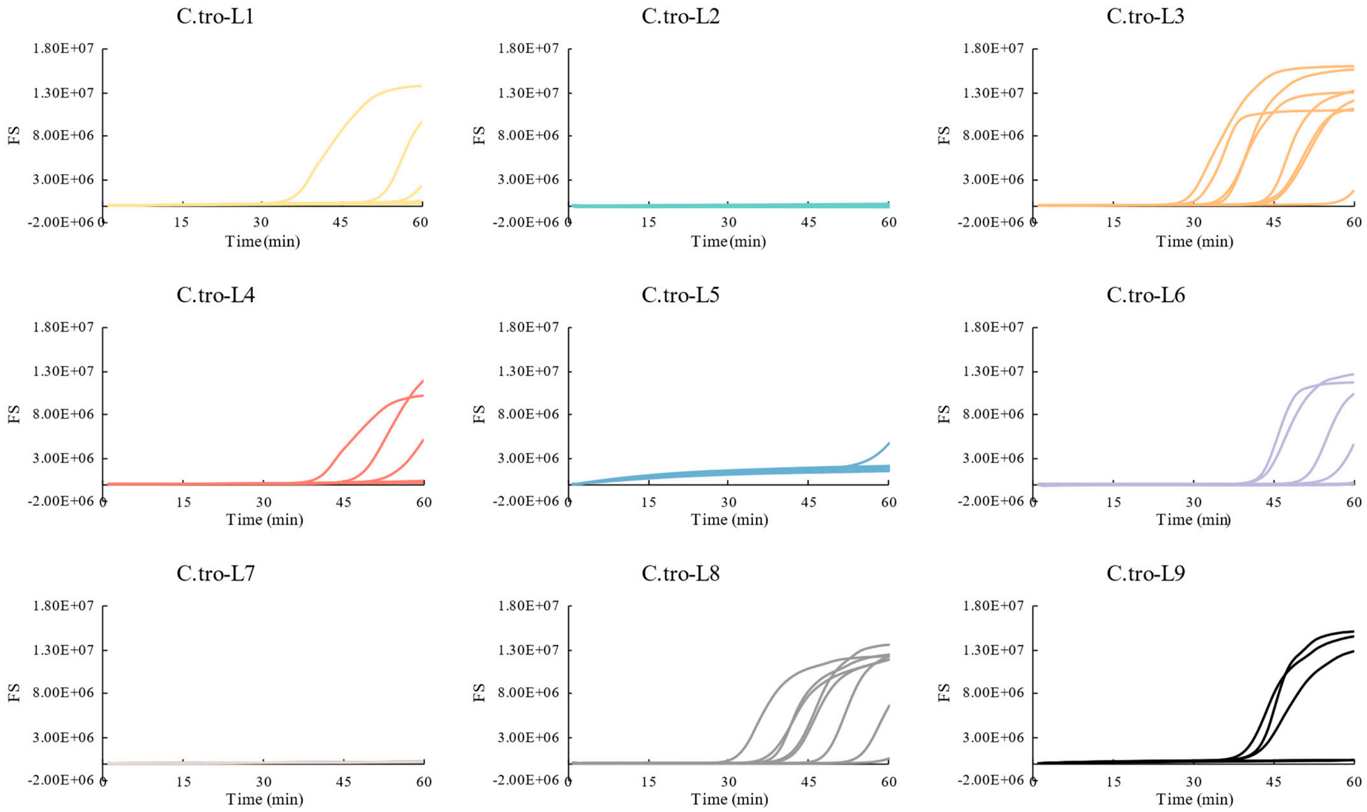

Figure S4. Selection of specificity of C.tro-L1 to C.tro-L9. In eight independent replicate runs of the 10 μL LAMP reaction without template at 65°C for 60 min, C.tro-L1, Ctro-L3 to C.tro-L6, C.tro-L8, and C.tro-L9 produced positive results and were eliminated, while the results of C.tro-L2 and C.tro-L7 were all negative, so the latter two sets of primers were selected for the next step. C.tro-L1~L9 were candidates for the screening of the LAMP primer sets used for the identification of *C. tropicalis*. FS, fluorescence signal.

Step 2: Selection of sensitivity. To select primers with high sensitivity, the LAMP reaction was performed in a 10 μL volume at 65°C for 40 min, with a 1 μL serially diluted (10^3 to 10^1 copies/μL) target template. In three independent replicate runs with a template of a certain dilution series, primers yielding positive results were selected for the next step (Figure S5).

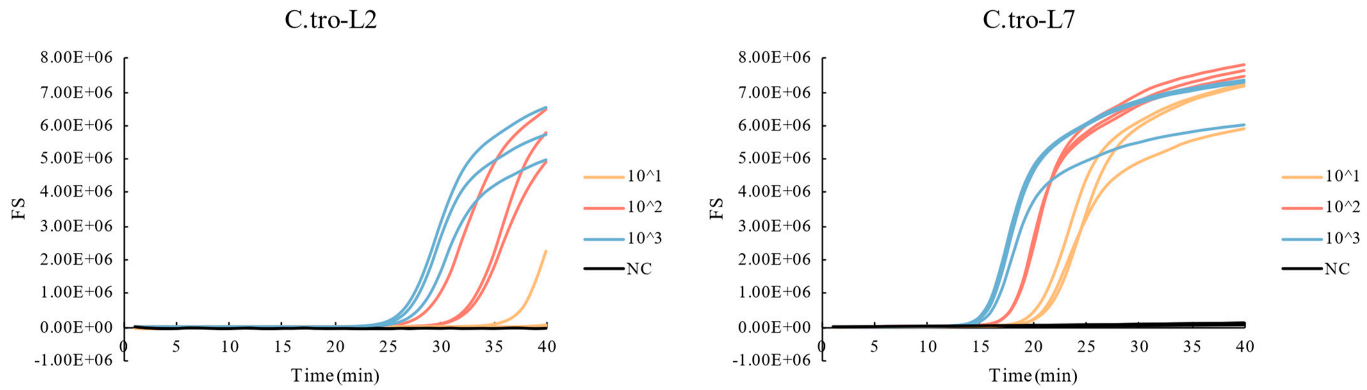

Figure S5. Selection of sensitivity of C.tro-L2 and C.tro-L7. Here, 10^n refers to the targeted template of 10^n copies/μL (n=1, 2, 3). NC refers to the negative control. FS refers to fluorescence signal. In three independent replicate runs of the 10 μL LAMP reaction with 1 μL targeted template of 10^2 copies/μL at 65°C for 40 min, the results of C.tro-L2 and C.tro-L7 were all positive, so the two sets of primers were selected for the next step.

Step 3: Verification of sensitivity. To verify the sensitivity of the primers selected above, the LAMP reactions were performed in a 10 μL volume at 65°C for 40 min, with 1 μL template of the dilution series according to the results of

Step 2. In 20 independent replicate runs with a template of the dilution series, primers that yielded positive results in at least 15 runs were selected for the next step (Figure S6).

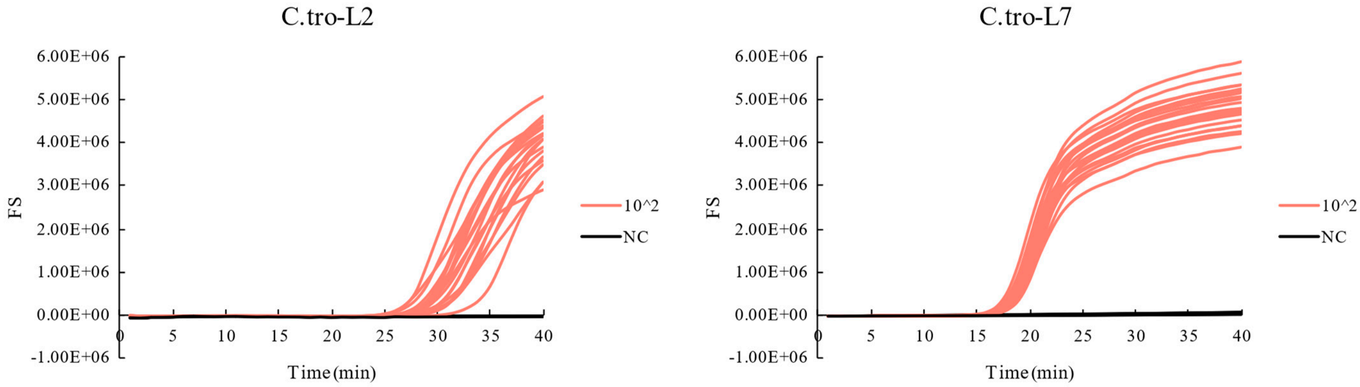

Figure S6. Verification of sensitivity of C.tro-L2 and C.tro-L7. Here, 10^2 refers to the targeted template of 10^2 copies/μL. NC refers to the negative control. FS refers to the fluorescence signal. In 20 independent replicate runs of the 10 μL LAMP reaction with 1 μL targeted template of 10^2 copies/μL at 65°C for 40 min, the results of C.tro-L2 and C.tro-L7 were all positive, so the two sets of primers were selected for the next step.

Step 4: Verification of secondary structure. To verify that the primers selected above did not easily produce artifacts (such as primer dimers), the LAMP reaction was performed in a 10 μL volume without template at 65°C for 40 min. In 92 independent replicate runs, primers that yielded negative results in at least 89 runs (numbers of false-positive results ≤ 3) were selected for the next step (Figure S7).

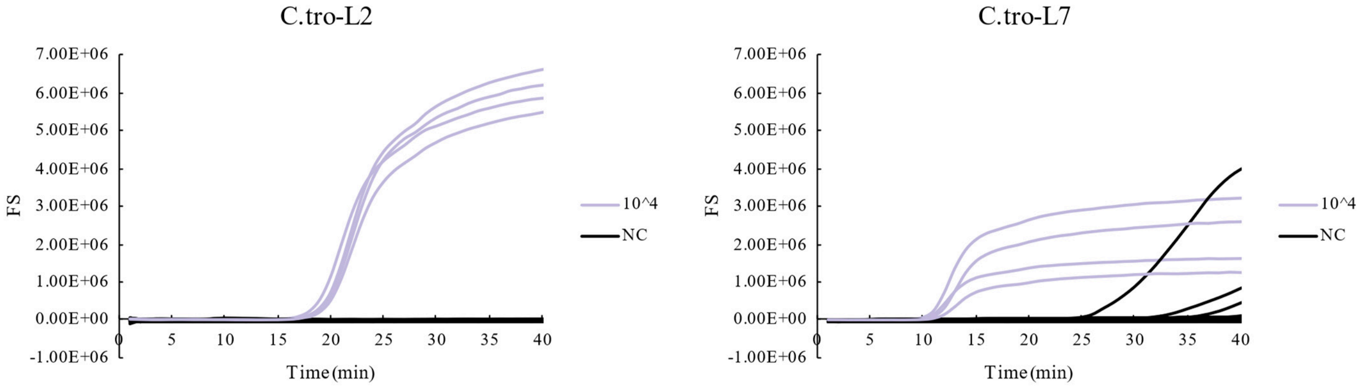

Figure S7. Verification of the secondary structure of C.tro-L2 and C.tro-L7. Here, 10^4 refers to the positive control with a targeted template of 10^4 copies/μL. NC refers to negative reaction without template. FS refers to the fluorescence signal. In 92 independent replicate runs of the 10 μL LAMP reaction without template at 65°C for 40 min, the results of C.tro-L2 were all negative, and the results of C.tro-L7 were negative in 89 runs, so the two sets of primers were selected for the next step.

Step 5: Verification of specificity. To verify the specificity of the primers selected above, the cross-reaction of LAMP was performed in a 10 μL volume at 65°C for 40 min, with a 1 μL template of 10^5 copies/μL of *C. albicans*, *C. glabrata*, *C. tropicalis*, *C. parapsilosis*, and *C. krusei*. In three independent replicate runs with each template, primers that yielded positive results only with the targeted template were selected (Figure S8).

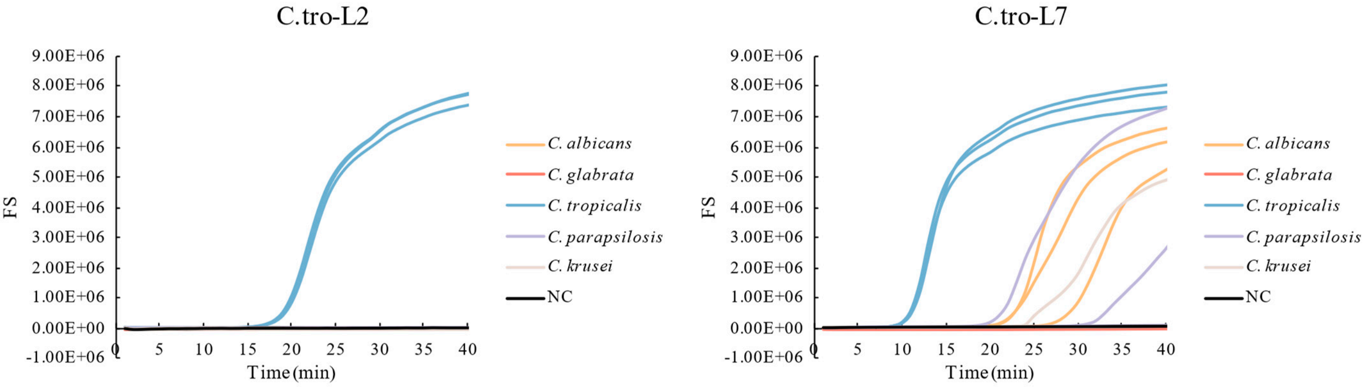

Figure S8. Verification of specificity of C.tro-L2 and C.tro-L7. NC refers to the negative control. FS refers to the fluorescence signal. In three independent replicate runs of the 10 μL LAMP reaction with each 1 μL template of 10^5 copies/μL at 65°C for 40 min, C.tro-L7 produced positive results with a targeted and non-targeted template and was eliminated, while C.tro-L2 produced positive results only with the targeted template and was ultimately selected.

Table S2. Screening results of each step of the 'five-step screening' method

Target	Designed primers	Step 1: selection of specificity	Step 2: selection of sensitivity (copies/μL)	Step 3: verification of sensitivity (copies/μL)	Step 4: verification of secondary structure	Step 5: verification of specificity
C. albicans	C.alb-L1	NC (7/8)	/	/	/	/
	C.alb-L2	NC (2/8)	/	/	/	/
	C.alb-L3	NC (2/8)	/	/	/	/
	C.alb-L4	NC (0/8)	10^2 (1/3)	/	/	/
	C.alb-L5	NC (4/8)	/	/	/	/
	C.alb-L6	NC (2/8)	/	/	/	/
	C.alb-L7	NC (0/8)	10^2 (0/3)	/	/	/
	C.alb-L8	NC (3/8)	/	/	/	/
	C.alb-L9	NC (0/8)	10^2 (0/3)	/	/	/
	**C.alb-L10**	NC (0/8)	10^2 (3/3)	10^2 (15/20)	NC (0/92)	√
C. glabrata	C.gla-L1	NC (0/8)	10^2 (1/3)	/	/	/
	C.gla-L2	NC (0/8)	10^2 (2/3)	/	/	/
	C.gla-L3	NC (2/8)	/	/	/	/
	C.gla-L4	NC (0/8)	10^2 (3/3)	10^2 (20/20)	NC (0/92)	×
	C.gla-L5	NC (7/8)	/	/	/	/
	C.gla-L6	NC (0/8)	10^2 (0/3)	/	/	/
	C.gla-L7	NC (0/8)	10^2 (0/3)	/	/	/
	C.gla-L8	NC (3/8)	/	/	/	/
	**C.gla-L9**	NC (0/8)	10^2 (3/3)	10^2 (20/20)	NC (0/92)	√
	C.gla-L10	NC (2/8)	/	/	/	/
C. parapsilosis	C.par-L1	NC (8/8)	/	/	/	/
	C.par-L2	NC (0/8)	10^2 (0/3)	/	/	/
	C.par-L3	NC (0/8)	10^2 (0/3)	/	/	/
	C.par-L4	NC (8/8)	/	/	/	/
	C.par-L5	NC (0/8)	10^2 (0/3)	/	/	/
	C.par-L6	NC (8/8)	/	/	/	/
	C.par-L7	NC (3/8)	/	/	/	/
	C.par-L8	NC (0/8)	10^2 (0/3)	/	/	/
	C.par-L9	NC (7/8)	/	/	/	/
	C.par-L10	NC (0/8)	10^2 (0/3)	/	/	/
	C.par-L11	NC (8/8)	/	/	/	/
	**C.par-L12**	NC (0/8)	10^2 (3/3)	10^2 (17/20)	NC (1/92)	√
C. tropicalis	C.tro-L1	NC (3/8)	/	/	/	/
	**C.tro-L2**	NC (0/8)	10^2 (3/3)	10^2 (20/20)	NC (0/92)	√
	C.tro-L3	NC (8/8)	/	/	/	/
	C.tro-L4	NC (3/8)	/	/	/	/
	C.tro-L5	NC (1/8)	/	/	/	/
	C.tro-L6	NC (4/8)	/	/	/	/
	C.tro-L7	NC (0/8)	10^2 (3/3)	10^2 (20/20)	NC (3/92)	×
	C.tro-L8	NC (7/8)	/	/	/	/
	C.tro-L9	NC (3/8)	/	/	/	/

NC refers to negative reactions without template;
10^n refers to positive reaction with targeted template of 10^n copies/μL;
(n/m) refers to n positive results in m independent replicate runs;
√ refers to the primer being selected through Step 5 (verification of specificity);
× refees to the primer being eliminated through Step 5 (verification of specificity);
The red-bolded primers were those ultimately selected through the whole process of primer selection. The green color refers to the primer being selected through that step. The red color refers to the primer being eliminated through that step.

Section 4: Validation of the performance of the sample-processing cassette

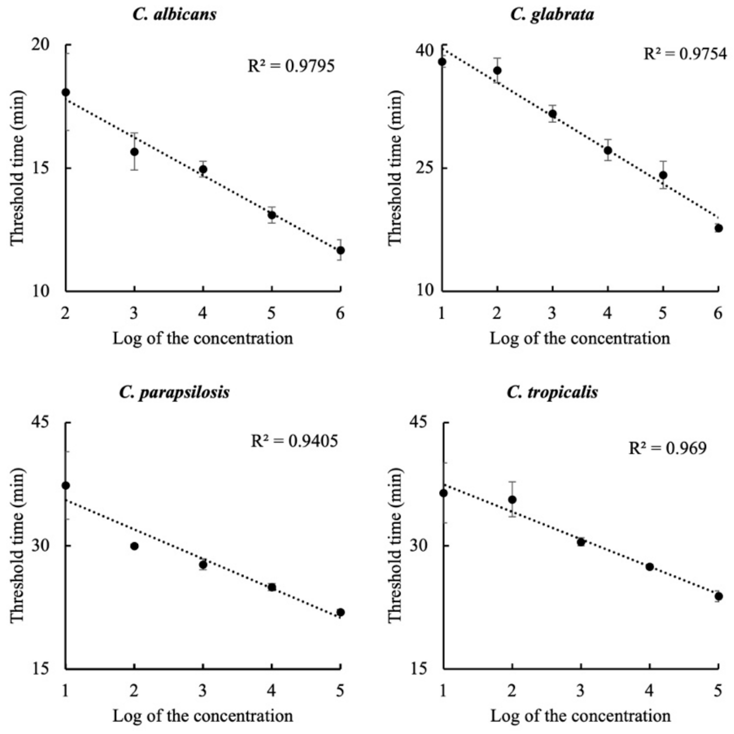

Figure S9. Detection sensitivity and linearity analysis of *Candida* species using the sample-processing cassette and LAMP assays. The coefficients of the concentration of *C. albicans*, *C. glabrata*, *C. parapsilosis*, and *C. tropicalis*, were 2.23, 8.10, 3.53, and 2.80, respectively. Log, logarithmic. Results were collected from three independent tests. Error bars indicate standard deviation from the mean.

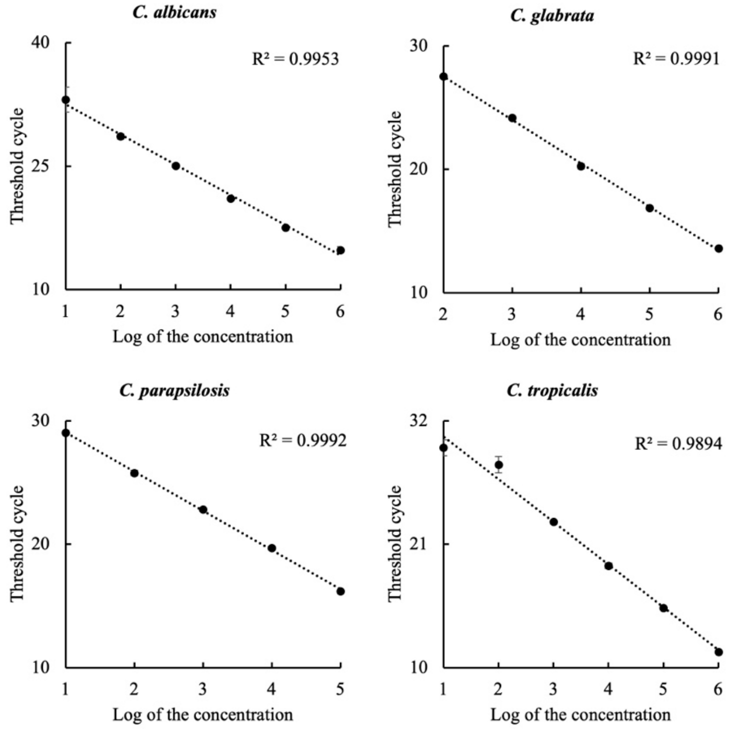

Figure S10. Detection sensitivity and linearity analysis of *Candida* species using a commercial kit and PCR assays. The coefficients of the concentration of *C. albicans*, *C. glabrata*, *C. parapsilosis*, and *C. tropicalis*, were 2.23, 8.10, 3.53, and 2.80, respectively. Log, logarithmic. Results were collected from three independent tests. Error bars indicate standard deviation from the mean.

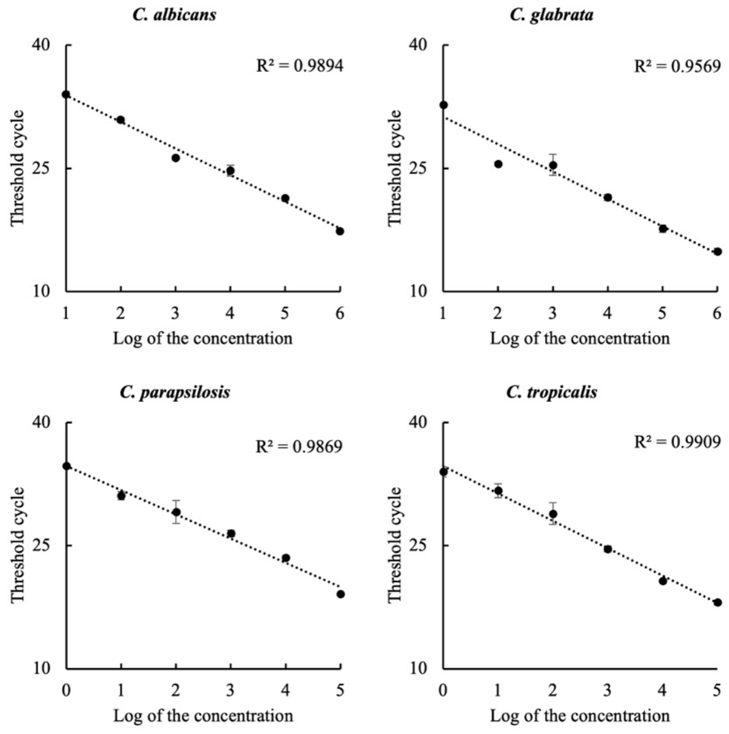

Figure S11. Detection sensitivity and linearity analysis of *Candida* species using the sample-processing cassette and PCR assays. The coefficients of the concentration of *C. albicans*, *C. glabrata*, *C. parapsilosis*, and *C. tropicalis*, were 2.23, 8.10, 3.53, and 2.80, respectively. Log, logarithmic. Results were collected from three independent tests. Error bars indicate standard deviation from the mean.

Section 5: Clinical sample results

Table S3. Diagnosis results and detection results of clinical samples

Sample No.	Sex	Age (year)	Clinical Diagnostics	RPT system assay results					PCR assay results				
				C.alb-L	C.gla-L	C.par-L	C.tro-L	Test result	C.alb-P	C.gla-P	C.par-P	C.tro-P	Test result
1	Female	29	BV	-	-	-	-	Negative	-	-	-	-	Negative
2	Female	30	VVC	+	-	-	-	*C. albicans*	+	-	-	-	*C. albicans*
3	Female	32	AV	-	-	-	-	Negative	-	-	-	-	Negative
4	Female	32	Normal	-	-	-	-	Negative	-	-	-	-	Negative
5	Female	37	VVC	-	-	+	-	*C. parapsilosis*	-	-	+	-	*C. parapsilosis*
6	Female	37	BV	-	-	-	-	Negative	-	-	-	-	Negative
7	Female	31	VVC	+	-	-	-	*C. albicans*	+	-	-	-	*C. albicans*
8	Female	50	Normal	-	-	-	-	Negative	-	-	-	-	Negative
9	Female	34	Normal	-	-	-	-	Negative	-	-	-	-	Negative
10	Female	33	VVC	+	-	-	-	*C. albicans*	+	-	-	-	*C. albicans*
11	Female	36	Normal	-	-	-	-	Negative	-	-	-	-	Negative
12	Female	39	Normal	-	-	-	-	Negative	-	-	-	-	Negative
13	Female	49	VVC	+	-	-	-	*C. albicans*	+	-	-	-	*C. albicans*
14	Female	33	VVC	-	+	-	-	*C. glabrata*	-	+	-	-	*C. glabrata*
15	Female	63	Normal	-	-	-	-	Negative	-	-	-	-	Negative
16	Female	30	VVC	-	-	-	+	*C. tropicalis*	-	-	-	+	*C. tropicalis*
17	Female	40	Normal	-	-	-	-	Negative	-	-	-	-	Negative
18	Female	31	AV	-	-	-	-	Negative	-	-	-	-	Negative
19	Female	40	VVC	+	-	-	-	*C. albicans*	+	-	-	-	*C. albicans*
20	Female	37	VVC	+	-	-	-	*C. albicans*	+	-	-	-	*C. albicans*
21	Female	33	VVC	+	-	-	-	*C. albicans*	+	-	-	-	*C. albicans*
22	Female	24	VVC	-	-	-	+	*C. tropicalis*	-	-	-	+	*C. tropicalis*
23	Female	27	VVC	-	+	-	-	*C. glabrata*	-	+	-	-	*C. glabrata*

24	Female	35	VVC	+	-	-	-	*C. albicans*	+	-	-	-	*C. albicans*
25	Female	32	BV	-	-	-	-	Negative	-	-	-	-	Negative

Continued Table S3.

Sample No.	Sex	Age (year)	Clinical Diagnostics	RPT system assay results					PCR assay results				
				C.alb-L	C.gla-L	C.par-L	C.tro-L	Test result	C.alb-P	C.gla-P	C.par-P	C.tro-P	Test result
26	Female	41	VVC	+	-	-	-	*C. albicans*	+	-	-	-	*C. albicans*
27	Female	18	BV	-	-	-	-	Negative	-	-	-	-	Negative
28	Female	34	VVC	-	-	-	+	*C. tropicalis*	-	-	-	+	*C. tropicalis*
29	Female	32	AV	-	-	-	-	Negative	-	-	-	-	Negative
30	Female	35	VVC	-	+	-	-	*C. glabrata*	-	+	-	-	*C. glabrata*
31	Female	42	VVC	-	+	-	-	*C. glabrata*	-	+	-	-	*C. glabrata*
32	Female	30	VVC	-	+	-	-	*C. glabrata*	-	+	-	-	*C. glabrata*

BV, bacterial vaginosis; AV, aerobic vaginitis; VVC, vulvovaginal candidiasis. In this work, BV and AV samples were referred to as 'non-VVC' samples.

RPT system, rapid sample processing and testing system.

C.alb-L, C.gla-L, C.par-L, and C.tro-L represent the LAMP primer sets used for the LAMP assays of *C. albicans*, *C. glabrata*, *C. parapsilosis*, and *C. tropicalis*, respectively.

C.alb-P, C.gla-P, C.par-P, and C.tro-P represent the PCR primer sets used for the PCR assays of *C. albicans*, *C. glabrata*, *C. parapsilosis*, and *C. tropicalis*, respectively.

The green color refers to the negative results. The red color refers to the positive results.

Table S4. Original detection results of clinical samples

Sample No.	Sex	Age (year)	Clinical Diagnostics	RPT system assay results positive rate (N/3) and Tt mean (min)					PCR assay results positive rate (N/3) and Ct mean				
				C.alb-L	C.gla-L	C.par-L	C.tro-L	Test result	C.alb-P	C.gla-P	C.par-P	C.tro-P	Test result
1	Female	29	BV	0/3	0/3	0/3	0/3	Negative	0/3	0/3	0/3	0/3	Negative
2	Female	30	VVC	3/3, 18.38	0/3	0/3	0/3	*C.albicans*	3/3, 24.54	0/3	0/3	0/3	*C.albicans*
3	Female	32	AV	0/3	0/3	0/3	0/3	Negative	0/3	0/3	0/3	0/3	Negative
4	Female	32	Normal	0/3	0/3	0/3	0/3	Negative	0/3	0/3	0/3	0/3	Negative
5	Female	37	VVC	0/3	0/3	3/3, 27.64	0/3	*C.parapsilosis*	0/3	0/3	3/3, 20.96	0/3	*C.parapsilosis*
6	Female	37	BV	0/3	0/3	0/3	0/3	Negative	0/3	0/3	0/3	0/3	Negative
7	Female	31	VVC	3/3, 20.18	0/3	0/3	0/3	*C.albicans*	3/3, 22.86	1/3, 31.27	0/3	0/3	*C.albicans*
8	Female	50	Normal	0/3	0/3	0/3	0/3	Negative	0/3	0/3	0/3	0/3	Negative
9	Female	34	Normal	0/3	0/3	0/3	0/3	Negative	0/3	0/3	0/3	0/3	Negative
10	Female	33	VVC	3/3, 15.64	0/3	0/3	0/3	*C.albicans*	3/3, 20.03	0/3	0/3	0/3	*C.albicans*
11	Female	36	Normal	0/3	0/3	0/3	0/3	Negative	0/3	0/3	1/3, 32.99	0/3	Negative
12	Female	39	Normal	0/3	0/3	0/3	0/3	Negative	0/3	0/3	0/3	0/3	Negative
13	Female	49	VVC	3/3, 14.72	0/3	0/3	0/3	*C.albicans*	3/3, 17.97	0/3	0/3	0/3	*C.albicans*
14	Female	33	VVC	0/3	3/3, 33.05	0/3	0/3	*C.glabrata*	0/3	3/3, 27.59	0/3	0/3	*C.glabrata*
15	Female	63	Normal	0/3	0/3	0/3	0/3	Negative	0/3	0/3	0/3	0/3	Negative
16	Female	30	VVC	0/3	0/3	0/3	3/3, 20.42	*C. tropicalis*	0/3	0/3	0/3	3/3, 25.37	*C. tropicalis*

17	Female	40	Normal	0/3	0/3	0/3	0/3	Negative	0/3	0/3	0/3	0/3	Negative
18	Female	31	AV	0/3	0/3	0/3	0/3	Negative	0/3	0/3	0/3	0/3	Negative
19	Female	40	VVC	3/3, 17.17	0/3	0/3	0/3	*C.albicans*	3/3, 17.75	0/3	0/3	0/3	*C.albicans*
20	Female	37	VVC	3/3, 20.06	0/3	0/3	0/3	*C.albicans*	3/3, 22.61	0/3	0/3	0/3	*C.albicans*

Continued Table S4.

Sample No.	Sex	Age (year)	Clinical Diagnostics	RPT system assay resuts positive rate (N/3) and Tt mean (min)					PCR assay results positive rate (N/3) and Ct mean				
				C.alb-L	C.gla-L	C.par-L	C.tro-L	Test result	C.alb-P	C.gla-P	C.par-P	C.tro-P	Test result
21	Female	33	VVC	3/3, 23.14	0/3	0/3	0/3	*C.albicans*	3/3, 24.64	0/3	0/3	0/3	*C.albicans*
22	Female	24	VVC	0/3	0/3	0/3	3/3, 19.59	*C. tropicalis*	0/3	0/3	0/3	3/3, 23.82	*C. tropicalis*
23	Female	27	VVC	0/3	3/3, 28.49	0/3	0/3	*C.glabrata*	0/3	3/3, 23.15	0/3	0/3	*C.glabrata*
24	Female	35	VVC	3/3, 18.23	0/3	0/3	0/3	*C.albicans*	3/3, 21.18	0/3	0/3	0/3	*C.albicans*
25	Female	32	BV	0/3	0/3	0/3	0/3	Negative	0/3	0/3	0/3	0/3	Negative
26	Female	41	VVC	3/3, 15.87	0/3	0/3	0/3	*C.albicans*	3/3, 18.93	0/3	0/3	0/3	*C.albicans*
27	Female	18	BV	0/3	0/3	0/3	1/3, 37.13	Negative	0/3	1/3, 29.79	0/3	0/3	Negative
28	Female	34	VVC	0/3	0/3	0/3	3/3, 25.11	*C. tropicalis*	0/3	0/3	0/3	3/3, 31.95	*C. tropicalis*
29	Female	32	AV	0/3	0/3	0/3	0/3	Negative	0/3	0/3	0/3	0/3	Negative
30	Female	35	VVC	0/3	3/3, 26.92	0/3	0/3	*C.glabrata*	0/3	3/3, 27.72	0/3	0/3	*C.glabrata*

31	Female	42	VVC	0/3	3/3, 27.85	0/3	0/3	*C.glabrata*	0/3	3/3, 28.96	0/3	0/3	*C.glabrata*
32	Female	30	VVC	0/3	3/3, 28.36	0/3	0/3	*C.glabrata*	0/3	3/3, 27.10	0/3	0/3	*C.glabrata*

Tt, threshold time; Ct, threshold cycle.

BV, bacterial vaginosis; AV, aerobic vaginitis; VVC, vulvovaginal candidiasis. In this work, BV and AV samples were referred to as 'non-VVC' samples.

RPT system, rapid sample processing and testing system.

C.alb-L, C.gla-L, C.par-L, and C.tro-L represent the LAMP primer sets used for the LAMP assays of *C. albicans*, *C. glabrata*, *C. parapsilosis*, and *C. tropicalis*, respectively.

C.alb-P, C.gla-P, C.par-P, and C.tro-P represent the PCR primer sets used for the PCR assays of *C. albicans*, *C. glabrata*, *C. parapsilosis*, and *C. tropicalis*, respectively.

The green color refers to the negative results. The red color refers to the positive results.

The yellow color refers to the false-positive results. As the false-positive rate was one-third, it was recognized as a negative detection result for the corresponding targets.
